# Connecting the dots: microstructural properties of white matter hyperintensities predict longitudinal cognitive changes in ageing

**DOI:** 10.3389/fnagi.2025.1520069

**Published:** 2025-02-19

**Authors:** Michael Courtney, Daniel Carey, Stephen Murphy, Silvin Knight, James F. Meaney, Rose Anne Kenny, Céline De Looze

**Affiliations:** ^1^Department of Radiology, St James’s Hospital, Dublin, Ireland; ^2^The Thomas Mitchell Centre for Advanced Medical Imaging, St James’s Hospital, Dublin, Ireland; ^3^The Irish Longitudinal Study on Ageing, School of Medicine, Trinity College Dublin, Dublin, Ireland; ^4^The Mercer’s Institute for Successful Ageing (MISA), St James’s Hospital, Dublin, Ireland

**Keywords:** white matter hyperintensities, cognitive decline, aging, vascular dementia, cardiovascular risk factors, neuroimaging

## Abstract

This study investigates the relationship between white matter hyperintensities (WMHs) and longitudinal cognitive decline in older adults. Using data from The Irish Longitudinal Study on Ageing (TILDA), we examined WMH characteristics, including volume, location, and microstructural integrity, in a community-dwelling population of 497 individuals over a six-year period. WMHs were categorised into phenotypes based on their size, fractional anisotropy (FA), and mean diffusivity (MD), with subtypes for periventricular and deep white matter lesions. We hypothesised that larger, microstructurally compromised lesions would be associated with accelerated cognitive decline. We isolated 11,933 WMHs, with an average of 24 WMHs per individual. Of these lesions, 6,056 (51%) were classified as Low Volume – High FA, 3193 (27%) were classified as Low Volume – Low FA and 2684 (22%) were classified as High Volume, Low FA. Our findings demonstrate that high-volume, low FA deep (*p* = 0.05) and periventricular (*p* = 0.004) lesions were significantly linked to cognitive decline (*X* = 12.9, *p* = 0.004), whereas small periventricular lesions with near normal microstructural properties do not predict cognitive decline. These results suggest that distinct WMH phenotypes may serve as markers for differential risks of cognitive impairment, providing potential targets for early intervention in at-risk populations.

## Introduction

The association between white matter integrity and adverse brain health outcomes is well-established. Increased white matter lesion burden has consistently been linked to higher risk of stroke, cognitive impairment, dementia, and mortality in cross-sectional and longitudinal studies involving diverse patient populations and healthy older adult cohorts (Hu et al., 2021; Debette and Markus, 2010; Herrmann et al., 2008). Assessment of white matter microstructural integrity using Diffusion Tensor Imaging (DTI) has revealed robust association with cognitive performance in both healthy older adults and various patient groups in cross-sectional and longitudinal designs (Prins et al., 2004; Debette et al., 2010; Prins and Scheltens, 2015). White matter hyperintensities (WMHs), initially localised to periventricular regions but progressing into deep white matter with increasing lesion number, play a crucial role in these associations.

WMH is a descriptive term used in MRI interpretation. A WMH is best described as a hyperintense lesion on T2/FLAIR sequencing in the cerebral white matter. These lesions are typically present in the periventricular white matter and deep cortical white matter. Typical age related vascular white matter hyperintensities are seen in the general population in approximately 10–20% of subjects aged 60 years old and with prevalence approaching 100% in persons aged 90 years and older (Smith et al., 2017). WMHs are reportedly common in the Japanese, Chinese, Caucasian, African-American and Caribbean Black demographics, increasing across all ethnicities with age (Zhuang et al., 2018). Typically, small WMHs in the elderly are asymptomatic, but these lesions can progress to large confluent lesions which can present with cognitive impairment, dementia, functional decline, urinary incontinence, balance and gait impairment and neuropsychiatric disorders (Misquitta et al., 2020).

While previous research has independently explored the impact of white matter macrostructure, microstructure, and spatial distribution on cognitive function, a comprehensive understanding of their cumulative and interconnected effects on cognitive decline is currently lacking. Previous investigations into the microstructural nature of WMHs, utilising fractional anisotropy (FA), mean diffusivity (MD), and magnetization transfer ratio (MTR), have demonstrated reduced white matter integrity in those with WMHs compared to those without (normal appearing white matter – NAWM). Specifically, FA and MTR values are significantly lower, while MD values are significantly higher in WMHs compared to NAWM (Bastin et al., 2009; Maniega et al., 2015). Histopathological studies suggest ischemia as the primary underlying process in the development of WMHs, but the process of microstructural damage is likely more complex and multifactorial (Pantoni and Simoni, 2003; Gouw et al., 2011; Moody et al., 1995; Poels et al., 2012).

Our hypothesis posits that heterogeneity in white matter integrity, as measured by MRI, may correlate with cognitive function. In this study, we categorise WMHs into six lesion phenotypes based on their location (periventricular or deep white matter), size (low/high volume), and microstructural properties (FA, MD). Using a large MRI sample of community-dwelling healthy older adults from The Irish Longitudinal Study on Ageing, a nationally-representative population-based study, we examined the effects of these phenotypes on cognitive decline over a six-year period. Our primary hypothesis is that due to a diverse combination of causative factors, WMHs manifest as variable phenotypes, and that different phenotypes are associated with cognitive decline. Specifically, we anticipated that large-volume deep white matter hyperintensities demonstrating significantly lower average FA and higher MD values are linked to accelerated cognitive decline over time. We believe that the characterisation of both macro- and microstructure of WHM lesions, and their spatial distribution, is critical for understanding their impact on cognitive decline and the associated risk of dementia.

The identification of WMH phenotypes associated with accelerated cognitive decline might impact clinical practice and aid in early detection of individuals at higher risk of progression from cognitive impairment to dementia, and could potentially lead to early implementation of intervention strategies which could improve patient outcomes.

## Methods

### Sample

**Figure 1:**
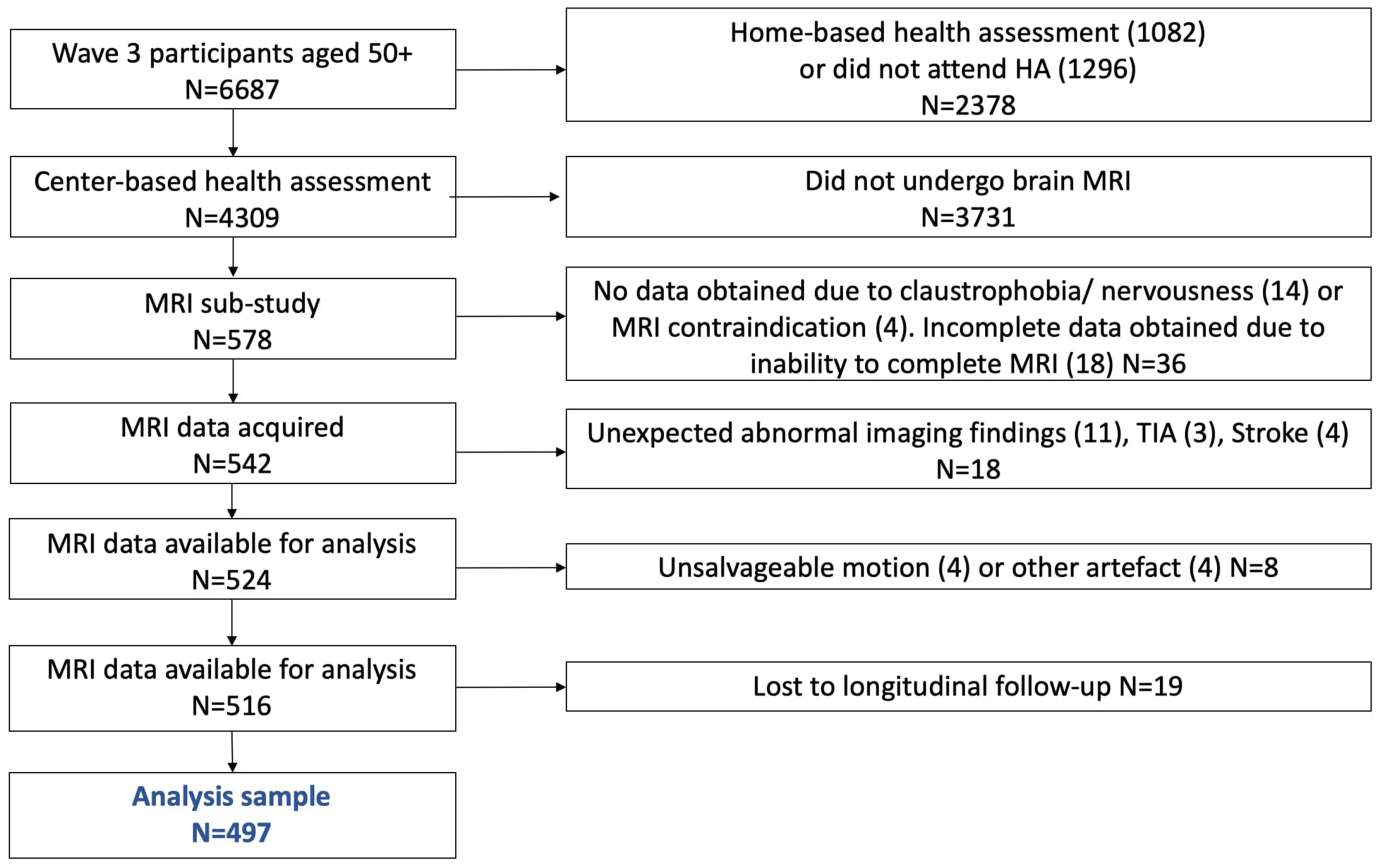
Study sample flowchart.

The Irish Longitudinal Study on Ageing (TILDA) is a large population-based study of a nationally-representative sample of community-dwelling older adults aged 50 years and over, resident in the Republic of Ireland. TILDA’s random sampling procedure and study design have been described elsewhere (Whelan and Savva, 2013). Briefly, participants have been followed biennially from Wave 1 (data collection baseline: 2009–2011; *N* = 8,504) and this analysis includes data from Wave 3 (2014–2015), Wave 4 (2016–2016), Wave 5 (2018–2018) and Wave 6 (2021–2021). Macro- and micro-structural measures of WMHs at Wave 3 and cognitive data (Wave 3–6) were available for 497 participants (see Figure 1 for a detailed breakdown). At each wave, participants took part in a computer assisted personal interview (CAPI) and completed a questionnaire (SCQ). At Wave 3, participants were also invited to take part in a health assessment and a sub sample of those who completed the health assessment were randomly selected to undergo magnetic resonance imaging (MRI). Ethical approval was granted by the Trinity College Faculty of Health Sciences Research Ethics Committee, Dublin, Ireland. Protocols conformed with the 1964 Declaration of Helsinki and its later amendments. Signed informed consent was obtained from all respondents prior to participation. Additional ethics approval was received for the MRI sub-study from the St James’s Hospital/Adelaide and Meath Hospital, Inc. National Children’s Hospital, Tallaght (SJH/AMNCH) Research Ethic Committee, Dublin, Ireland. Those attending for MRI were also required to complete an additional MRI-specific consent form.

### MRI data acquisition and processing

The MRI sampling procedure has been previously described in detail (De Looze et al., 2020). In brief, MRI scanning was completed at the Thomas Mitchell Centre for Advanced Medical Imaging (CAMI), St. James’ Hospital, Dublin, (3 T Philip’s Achieva system and 32-channel head coil). The study protocol included T1, fluid attenuation inversion recovery (FLAIR) and diffusion weighted imaging (DWI) sequences. T1-weighted scans were acquired using a 3D Magnetisation Prepared Rapid Gradient Echo (MP – RAGE) sequence (FOV (mm): 240 × 240 × 162; SENSE factor: 2; TR: 6.7 ms; TE: 3.1 ms; flip angle: 8°; voxel size (mm): 0.8 × 0.8 × 0.9). FLAIR images were acquired using a 2D turbo spin-echo sequence (FOV (mm): 230 × 230; TR: 11000 ms; TE: 125 ms; TI: 2800 ms; flip angle: 90°; number of slices: 30; slice thickness: 4 mm; slice gap: 1 mm; voxel size(mm): 0.45 × 0.45). DWI images were acquired using a high spatial (1.9 mm^3^) and high angular resolution (61-direction) protocol with a high diffusion sensitivity (*b* = 1,200 s/mm^2^; TR: 2885 ms; TE: 64 ms; flip angle: 90°; number of slices: 30; slice thickness: 4 mm; slice gap 1 mm: voxel size (mm): 0.96 × 0.96).

Images were pre-processed using SPM12 (University College London, London, UK) and ExploreDTI^1^ (Leemans et al., 2009). All images were reorientated to a canonical SPM template. The dataset was inspected for appropriate orientation and any gross artifacts before processing and segmentation. DWI preparation included correcting multiple prerequisite parameters in the ExploreDTI software toolbox for MATLAB.^2^ The entire standardised dataset was converted to ExploreDTI compatible diffusion files, corrected for signal drift (Vos et al., 2017), Gibbs Ringing artefact (Perrone et al., 2015), head motion and eddy current artefact with registration to the B0 reference image (Soares et al., 2013) and EPI deformation correction via registration to the T1 sequence (Irfanoglu et al., 2012). These commands were performed using the native ExploreDTI toolbox plugins.

### Location, size and microstructural properties of WMHs

White matter hyperintensities (WMHs) were segmented utilising the Lesion Prediction Algorithm (LPA) (Schmidt, 2016), implemented in the LST toolbox version 3.0.0 for SPM12.^3^ The LPA was applied to both FLAIR and T1 images. Meticulous visual image review ensured accurate and appropriate segmentation of only WMHs. Each segmented lesion received a unique lesion number, and an individualised atlas of lesions was created for each subject. To objectively localise each lesion, the SPM Anatomic Automatic Labeling extension was employed (Rolls et al., 2015). Lesions were categorised based on their anatomical position in the periventricular or the deep white matter. Periventricular lesions were defined as those within 15 mm of a ventricle (Nyquist et al., 2015), while deep white matter lesions were those outside this range. Atlas-based tractography was performed using the segmented lesions as regions of interest in each subject, utilising the native ExploreDTI plugin, and diffusion metrics (FA and MD) unique to each WMH were calculated.

### WMHs phenotypes

Lesions were initially clustered based on both macrostructural properties (volume in mm^3^) and microstructural properties (FA and MD) using k-means clustering (cluster package in R version 4.0.3 with RStudio Version 1.4.1103). The optimal number of lesion phenotypes or clusters was determined using the elbow method. Three primary lesion phenotypes were identified. These phenotypes were further stratified based on their location (deep vs. periventricular), resulting in a total of six lesion phenotypes. The frequency of each lesion phenotype per individual was calculated and used for subsequent analysis.

### Cognitive function

Global cognitive function at each wave [Wave 3 to 6; MEAN ± SD-year follow-up; wave 3–28.8 (1.5), wave 4–28.6 (1.3) wave 5–27.9 (1.7) and wave 6–27.6 (1.8)] was assessed using a battery of neuropsychological tests, including 10-word immediate recall test (2 trials), 10-word delayed recall test, semantic verbal fluency (animal naming), and orientation (date, day, month, year). A composite score per participant and at each wave was derived by combining individual test scores to broadly reflect global cognitive function. The composite scores were generated based on an equally unit-weighted approach using standardised scores (*z* scores). The lower the composite score, the worse the cognitive function.

### Covariates

Covariates included age, sex, education (none/primary, secondary, or tertiary/higher); smoking (never, past or current); problematic alcohol consumption (CAGE questionnaire; yes or no); physical activity (International Physical Activity Questionnaire – IPAQ; short-form; low, moderate or high); body mass index (BMI, continuous variable); pre-existing self-reported physician-diagnosed cardiovascular diseases and events (CVDEs, none or one or more of the following: angina, heart attack, congestive heart failure, stroke, transient ischemic attack, and atrial fibrillation); average systolic and diastolic blood pressure measurements obtained twice using an OMRON digital automatic blood pressure monitor (Model M10-IT); antihypertensive medication [Anatomical Therapeutic Chemical (ATC) codes C02 (antiadrenergic agents), C03 (diuretics), C07 (*β* blockers), C08 (calcium-channel blockers), and C09 (angiotensin- converting enzyme inhibitors)]; antidepressant medication (ATC code N06A); and symptoms of depression (the Centre for Epidemiological Studies Depression Scale – CESD; continuous variable).

### Statistical analysis

All statistical analyses were performed using R version 4.0.3 with RStudio Version 1.4.1103. The observed sample was first characterised using unadjusted means and standard deviations for continuous variables and percentages (%) for categorical variables. To estimate selection bias, the observed sample was compared to the total cohort including those excluded from analysis. Independent t-tests and chi-squared tests where appropriate were used to assess differences between the two groups. Linear mixed effect models were used to examine the relationship between lesion phenotypes (predictor) and cognitive function at waves 3, 4, 5 and 6 (outcome). Fixed effects included the number of lesion phenotypes in separate models and waves with an interaction term. The significance of the interaction was assessed using likelihood ratio tests. Participants constituted the random intercept. Baseline models included age, sex and education as covariates; further adjustment included smoking, alcohol consumption, physical activity, BMI, CVDEs, average systolic and diastolic blood pressure, depressive symptoms and antihypertensive and antidepressant medication.

## Results

### Data description

Table 1 provides descriptive statistics of the study sample (*n* = 497) at Wave 3. Mean age of the study sample was 68.5 years (SD 7.56); 51.9% of the participants were female and 20.7% had a low (primary school) level of education. Mean MMSE (Mini Mental State Examination) at Wave 3 was 28.8 (SD = 1.5).

**Table 1:** Demographic descriptive statistics of the sample population.

Descriptor	*N* = 497
Age (mean, sd)	68.5 (7.5)
Sex (Female, %)	51.9
Education (Low, %)	20.7
CVD conditions (>1, %)	29.7
Systolic/Diastolic BP (mean, sd)	134.3 (18.3)/79.9 (10.3)
Hypertension (on meds, %)	15.7
BMI (mean, sd)	28.0 (4.3)
Smoking (Present, %)	6.8
Physical activity (low, %)	33.8
Problematic alcohol (%, missing %)	8.8, 11.2
Depression symptoms (mean, sd)	3.5 (3.4)

A total of 11,933 individual white matter lesions were segmented and analysed from the cohort (*n* = 497) (Table 2). On average, each participant exhibited 24 white matter hyperintensities (WMHs) with a mean volume of 263.21 mm^3^.

**Table 2:** Descriptive statistics of the sample population’s segmented white matter hyperintensities and cognitive testing.

Descriptor	*N* = 497
Number of White Matter Lesions (mean (sd), [min, max])	24.0 (12.6), [4,141]
Lesion Volume mm^3^ (mean (sd), [min, max])	263.21 (1333.0), [0.72, 34653.58]
Lesion average FA (mean (sd), [min, max])	0.29 (0.10), [0.04, 0.78]
Lesion average MD (10^3) (mean (sd), [min, max])	1.10 (0.35) [0.36, 2.94]
Lesion average RD (10^3) (mean (sd), [min, max])	0.94 (0.34), [0.26, 2.83]
Lesion average AD (10^3) (mean (sd), [min, max])	1.43 (0.40), [0.47, 3.31]
Location (Deep, %)	14.6
MMSE (mean, sd)	28.8 (1.5)
Immediate Recall, 2 trials (mean, sd)	14.2 (2.9)
Delayed Recall (mean, sd)	6.2 (2.4)
Animal Naming (mean, sd)	19.4 (5.4)

**Figure 2:**
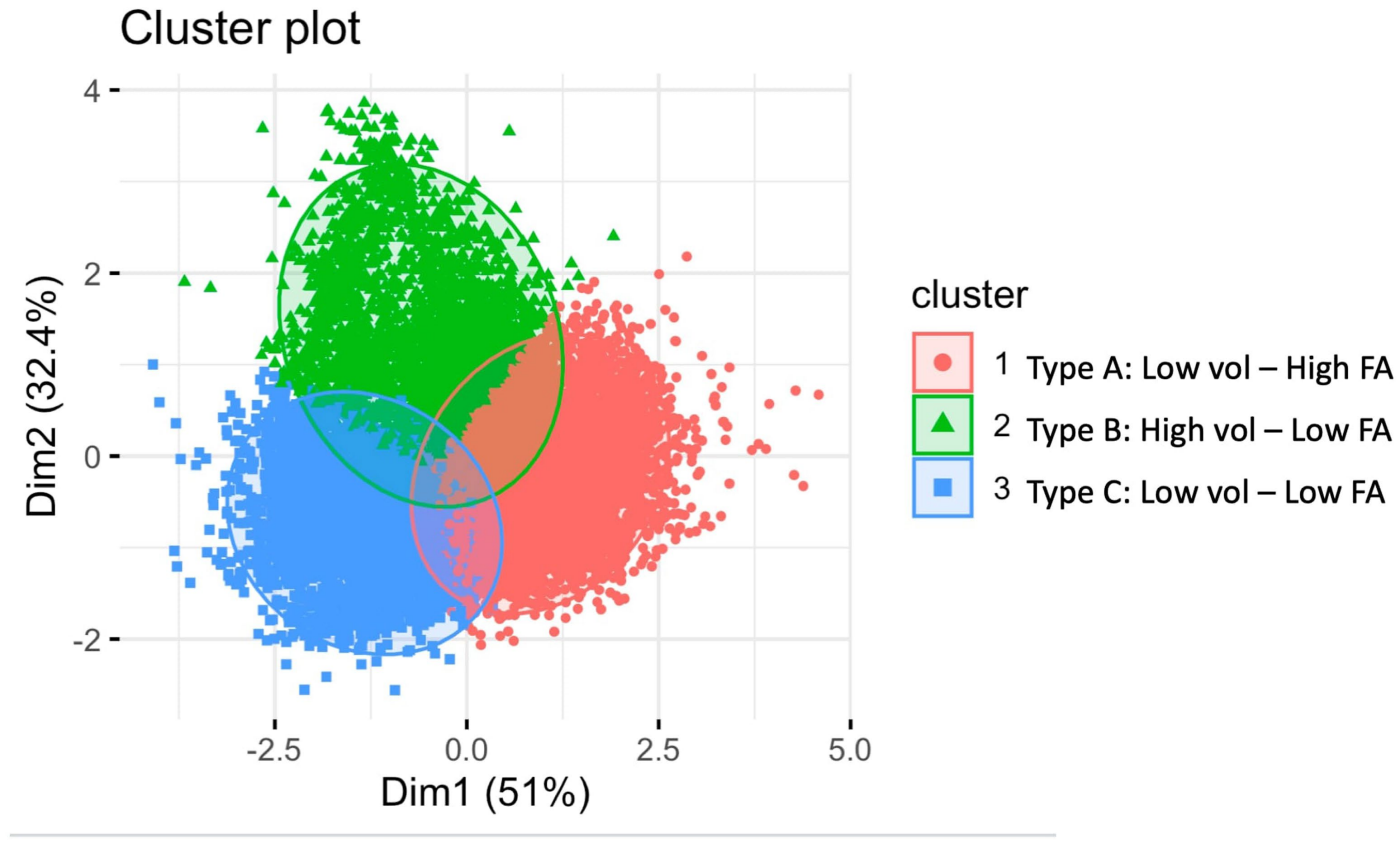
Cluster plot of white matter hyperintensities.

Cluster analyses revealed three distinct lesion phenotypes (Figure 2): Type A (Low Volume, High FA/Low MD), Type B (Low Volume, Low FA/High MD) and Type C (High Volume, Low FA/High MD), and, each further categorised into periventricular and deep white matter lesions. We will omit the MD descriptor for simplicity from this point. 40% of lesions were Periventricular Type A (Low Volume, High FA), 26% were Periventricular Type B (Low Volume, Low FA), 19% were Periventricular Type C (High Volume, Low FA), 11% were Deep Type A (Low Volume, High FA), 3% were Deep Type C (High Volume, Low FA), and 1% were Deep Type B (Low Volume, Low FA). Descriptive characteristics of these six lesion phenotypes are presented in Table 3.

**Table 3:** Lesion phenotypes and descriptive characteristics.

Characteristic	Low volume-high FA (*N* = 6,056)		Low volume-low FA (*N* = 3,193)		High volume-low FA (*N* = 2,684)	
Locations	Deep (*N* = 1,295) (11%)	PVT (*N* = 4,761) (40%)	Deep (*N* = 119) (1%)	PVT (*N* = 3,074) (26%)	Deep (*N* = 336) (3%)	PVT (*N* = 2,348) (19%)
Volume (mean, sd)	24.7 (23.0)	24.4 (28.1)	20.9 (18.6)	28.6 (41.1)	248.6 (623.2)	1200.4 (2806.0)
Average FA (mean, sd)	0.30 (0.07)	0.35 (0.09)	0.16 (0.05)	0.20 (0.07)	0.24 (0.05)	0.27 (0.07)
Average MD (mean, sd)^3	0.83 (0.00)	0.87 (0.00)	1.19 (0.00)	1.55 (0.00)	0.96 (0.00)	1.14 (0.00)
Average RD (mean, sd)^3	0.69 (0.00)	0.70 (0.00)	1.08 (0.00)	1.39 (0.00)	0.84 (0.00)	0.98 (0.00)
Average AD (mean, sd)^3	1.11 (0.13)	1.22 (0.24)	1.39 (0.42)	1.88 (0.41)	1.20 (0.13)	1.47 (0.21)
Age (mean, sd)	71.1 (7.5)	69.1 (7.6)	71.6 (6.8)	67.1 (7.7)	71.7 (7.3)	69.3 (7.1)
Sex (female, %)	56.2	46.9	57.9	46.3	62.2	49.5
Education (low, %)	27.3	22.2	21.0	19.8	32.4	23.2
CVD conditions (2+)	43.6	31.8	42.8	26.8	52.1	32.1
Systolic BP (mean, sd)	136.2 (17.8)	135.0 (17.5)	142.3 (17.2)	133.8 (18.4)	135.5 (17.5)	134.7 (17.8)
Hypertension (on meds, %)	22.5	17.4	31.9	14.8	27.6	16.4
BMI (mean, sd)	28.36 (4.4)	28.41 (4.2)	27.84 (4.5)	28.12 (4.3)	28.35 (4.6)	27.95 (4.3)
Smoking (current, %)	10.3	7.7	5.0	6.3	13.9	8.7
Problematic Alcohol (yes, %)	11.5	9.0	19.3	10.1	10.1	7.4
Physical Activity (low, %)	40.3	34.4	36.9	33.8	38.6	33.9
Depression (mean, sd)	3.56 (3.42)	3.37 (3.3)	3.06 (3.16)	3.46 (3.39)	3.94 (3.78)	3.56 (3.47)

Where SD values are 0.00, this means *p* < 0.001.

Comparisons among the lesion phenotypes, with the Periventricular low volume, high FA Type A (Low Volume, High FA) group as the reference, revealed notable demographic and clinical differences. Those with deep phenotypes (Type A, B, C) were older (*p* < 0.001), more likely to be women (Type A (Low Volume, High FA) and C (High Volume, Low FA); *p* < 0.001), and exhibited lower levels of education (Type A and C; *p* < 0.001). Individuals in the Deep Phenotype groups were more likely to have two or more cardiovascular conditions (Type A (Low Volume, High FA) and C (High Volume, Low FA); *p* < 0.001, Type B (Low Volume, Low FA) *p* < 0.05), higher systolic blood pressure (Type A (Low Volume, High FA) *p* < 0.05, Type B (Low Volume, Low FA) *p* < 0.001), current smoking habits (Type A (Low Volume, High FA); *p* < 0.001, Type C (High Volume, Low FA); *p* < 0.01), problematic alcohol consumption (Type A (Low Volume, High FA); *p* < 0.01, Type B (Low Volume, Low FA); *p* < 0.001), lower levels of physical activity (Type A (Low Volume, High FA); *p* < 0.001, Type C (High Volume, Low FA); *p* < 0.05), and higher levels of depressive symptoms (Type C (High Volume, Low FA); *p* < 0.01, Type A (Low Volume, High FA); *p* < 0.1). Similar trends were observed in the Periventricular Type B (Low Volume, Low FA) group.

### The association between lesion phenotypes and 6-year cognitive decline

**Figure 3:**
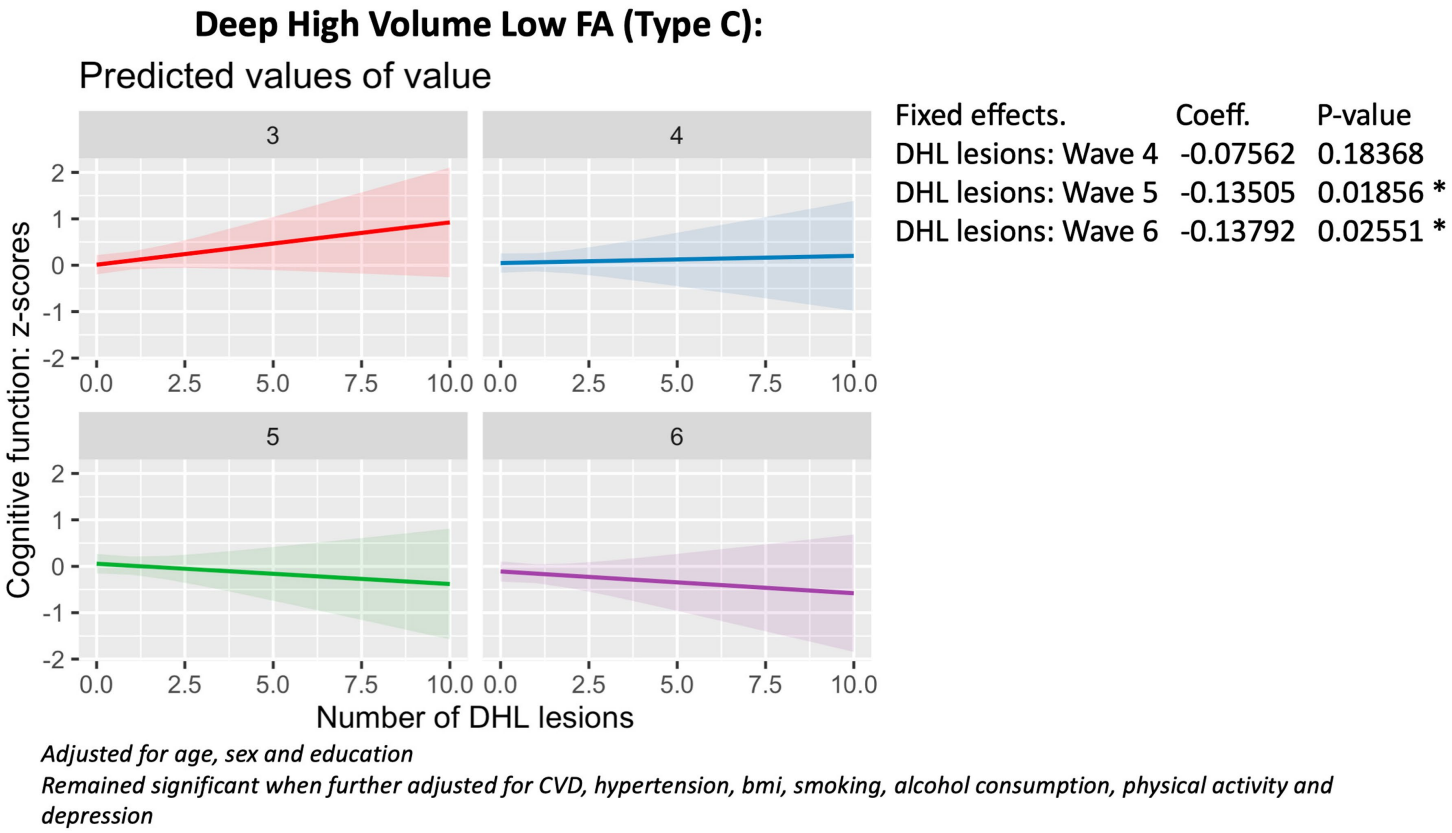
Association with cognitive function (wave 3 - wave 6), deep Type C lesions.

**Figure 4:**
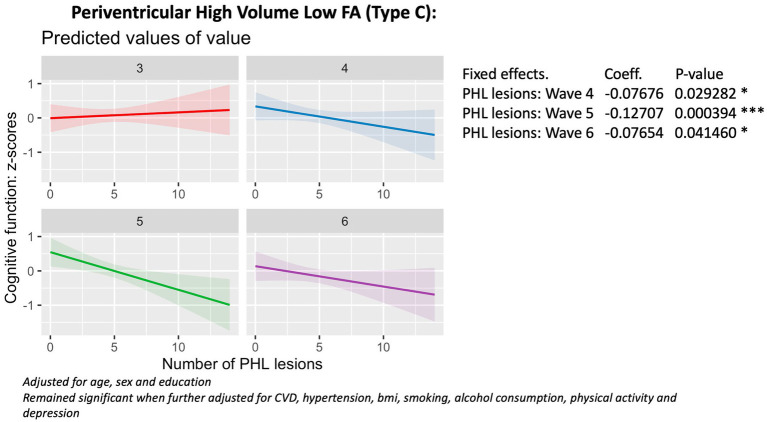
Association with cognitive function (wave 3 - wave 6), periventricular Type C lesions.

**Figure 5:**
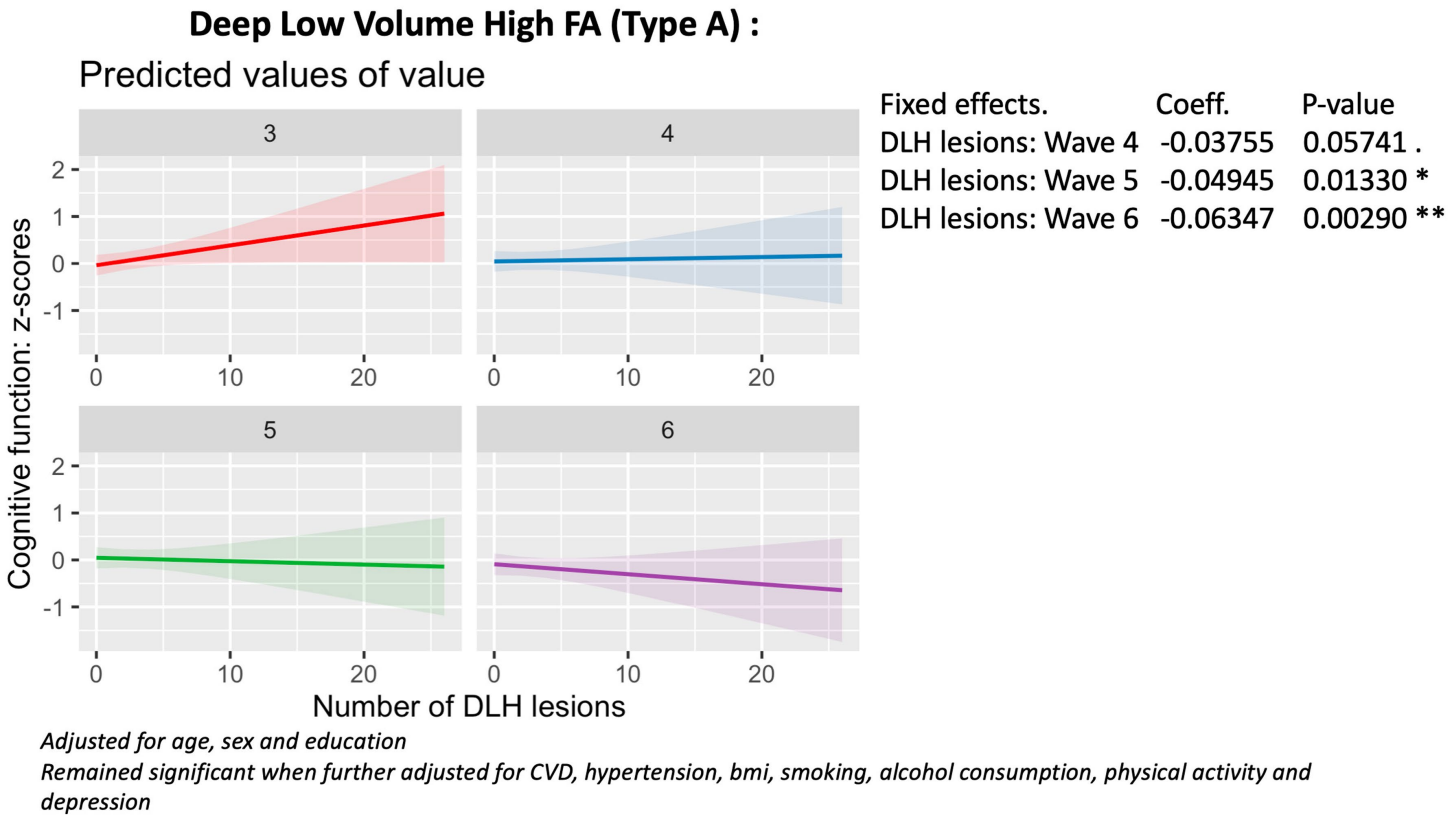
Association with cognitive function (wave 3 - wave 6), deep Type A lesions.

Baseline Linear Mixed Effects (LME) models revealed that a higher number of deep (*X* = 7.3; *p* = 0.05) and periventricular (*X* = 12.9, *p* = 0.004) high volume, low fractional anisotropy lesion phenotypes (Type C) were associated with accelerated cognitive decline from Wave 4 to Wave 6 (Figures 3, 4), compared to the reference group with periventricular low volume, high FA (Type A) lesions. Additionally, a higher number of deep low volume, high FA (Type A) lesions were also linked to accelerated cognitive decline (*X* = 10.5; *p* = 0.01) (Figure 5).

**Figure 6:**
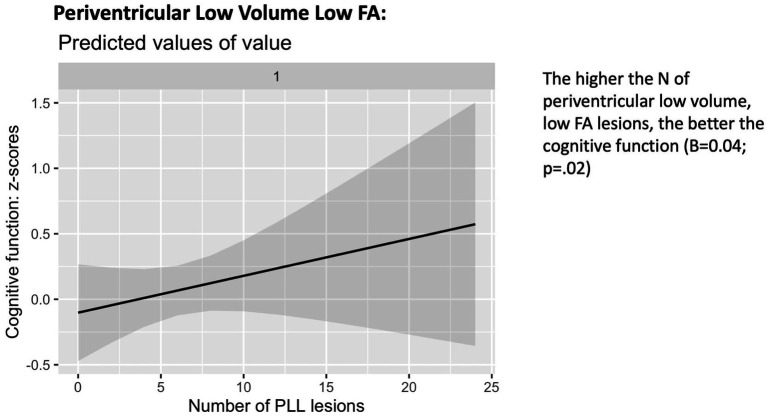
Association with cognitive function (wave 3 - wave 6), periventricular Type B lesions.

The phenotype by Wave correlation did not reach significance for the number of periventricular low volume, high FA (Type A) lesions, deep and periventricular low volume, low FA (Type B) lesions (*p* > 0.05). There were no significant associations between the number of periventricular Type A (low volume, high FA) lesions (*p* = 0.46) or deep Type B (low volume, low FA) lesions (*p* = 0.48) and cognitive decline. In contrast, the number of periventricular low volume, low FA (Type B) lesions was overall positively associated with cognitive function (*B* = 0.04; 95% CI = 0.00, 0.09; *p* = 0.02) (Figure 6).

These findings remained robust to adjustments for smoking, alcohol consumption, physical activity, body mass index (BMI), cardiovascular diseases (CVDEs), average systolic and diastolic blood pressure, antihypertensive and antidepressant medication, and of depression.

## Discussion

Our study sought to elucidate the combined impact of white matter hyperintensity (WMH) volume, microstructure, and location on cognitive decline over 6 years in a large MRI sample of community-dwelling older adults from a nationally representative population-based study of aging. Our hypothesis that WMHs manifest as variable phenotypes is supported by the identification of three potential groups of lesions based on MRI-defined macro and microstructural white matter characteristics. Additionally our analysis confirms that deep white matter, large volume hyperintensities with significantly lower average fractional anisotropy (FA) and higher mean diffusivity (MD) are associated with accelerated cognitive decline over time. We present a novel approach to classifying WMHs based on a combination of location, macro- and microstructural properties and describe the associations of these phenotypes with longitudinal cognitive decline.

Using a data-driven approach, we identified three distinct groups of lesions based on their macro and microstructural white matter characteristics (Low Volume/High FA, Low Volume/Low FA, and High Volume/Low FA). These groups were further stratified into periventricular and deep white matter lesions, resulting in six distinct WMH phenotypes. We found that WMHs characterised by low volume and relatively high average FA in the periventricular region, resembling normal appearing white matter microstructural properties were the most frequently identified phenotype.

Our observations highlight the relationship between MRI phenotypes and various demographic and clinical factors. Lesions in the deep white matter, as well as those of low volume and low FA in the periventricular white matter (Type B), are associated with increased age, female gender, lower education levels, higher cardiovascular risk factors, antihypertensive medication use, smoking, problematic alcohol use, and a sedentary lifestyle aligning with existing literature detailing the relationship between lifestyle, vascular disease, and brain health.

Deep WMHs Types A (low volume, high FA) and C (high volume, low FA) and Periventricular Type C (high volume, low FA) lesions were associated with accelerated cognitive decline over 6 years, indicating that lesions in the deep white matter and higher volume lesions, regardless of distribution, are more likely to be linked with cognitive decline. While this lesion-load dependent relationship aligns with existing literature, the observation that lesions located beyond the periventricular region in the deep white matter is poorly appreciated, providing potential insights into the significance of WMHs.

The most robust association with cognitive decline was found in Periventricular Type C lesions (high volume, low FA), representing lesions with architectural values furthest removed from normal appearing white matter (*p* = 0.004). This aligns with previous observations of decreased FA in various brain regions in individuals with mild cognitive impairment (Cherubini et al., 2010; Bai et al., 2009; Bozoki et al., 2012). Our findings support an association between microstructural damage and cognitive impairment, providing evidence for a lesion-burden dependent relationship.

Of note, we did not identify a subtype of WMH which demonstrated high volume and high FA, which suggests that as lesion size increases, microstructural integrity decreases. The absence of high-volume, high-FA lesions in our analysis precluded the opportunity to explore differential associations based on microstructural properties. The absence of these lesions suggests that as WMHs increase in volume, their microstructural integrity declines, as evidenced by reduced FA values. This finding supports the progressive and pathological nature of WMHs, underscoring that lesion growth likely correlates with progressive axonal disruption, loss of myelin integrity and white matter connectivity.

Our observation that Periventricular Type B (low volume, low FA) lesions appear to be protective against cognitive decline raises questions and invites a deeper exploration of the underlying vascular dynamics in the periventricular regions. The periventricular white matter, situated in proximity to the lateral ventricles, is known to be particularly vulnerable to changes in cerebral blood flow due to its unique vascular supply. The periventricular white matter is primarily nourished by small perforating arteries arising from larger vessels, such as the anterior, middle, and posterior cerebral arteries. These penetrating arteries course through the periventricular region, supplying the adjacent white matter.

The propensity for periventricular white matter ischemic insult is well established and multifactorial, culminating in white matter (ischaemic) lesions in the setting of vascular risk factors. The disposition of the arterial supply results in a watershed area in the periventricular white matter, which is highly sensitive to changes in perfusion pressure making it susceptible to hypoperfusion-related damage. Hypoxia has been described in the pathogenesis of WMHs using specific markers for vascular morphology and tissue hypoxia (Fernando et al., 2006). Increased vessel wall thickness and larger perivascular spaces have been observed in white matter disease (Moody et al., 2004). Particularly in the setting of deep white matter disease, capillary endothelial cells have been observed to be activated with increased arteriolar tuberosity and decreased vessel density (Brown et al., 2002).

These factors contribute to the underlying vulnerability of the periventricular white matter. The recognised mechanisms of damage to the periventricular white matter may be a slow or chronic process, that might allow the brain to adapt over time. A compensatory adaption could explain why some periventricular lesions do not significantly impact cognitive function. The periventricular regions are notably less densely packed neuronal pathways (Zatorre et al., 2012), therefore damage to the periventricular region may primarily affect less critical pathways. It remains unclear if small periventricular lesions can be classified as a normal part of the aging process, rather than a specific pathological hallmark and in isolation these may not correlate with significant cognitive impairment. Our finding relating to the protective nature of periventricular Type B lesions (low volume and low FA), may relate to factors not accounted in our cognitive testing combined with subjects compensatory mechanisms. As the affected periventricular area may not significantly impact global cognition, particularly when dealing with low volume lesions, there may be deficits in processing speed or executive function which are not captured in broad cognitive testing.

Our study draws parallels with a recent article by Li et al., which concludes that WMHs are non-specific lesions associated with amyloid-beta deposition, cognitive decline and multiple vascular risk factors (Li et al., 2024). Our findings are in line with Li’s findings in that we state, as already established in the literature, WMHs are more prevalent in subjects with two or more cardiovascular conditions, higher levels of systolic blood pressure, positive smoking history and lower levels of physical activity. However, while this recent article posits that WMHs are non-specific lesions in terms of underlying aetiology, we offer that WMHs may be defined as separate phenotypes, which can specifically serve as markers predictive of longitudinal cognitive decline, as we have shown that individuals who possess large volume lesions with diffusion metrics furthest removed from normal (i.e., Type C lesions with large volume and low FA), demonstrated accelerated cognitive decline over a 6 year period compared to others.

With regard to the underlying aetiology of WMHs, evidence in the literature has trended toward a prevalent vascular or ischaemic aetiology. Other factors have been described and likely play a role, though probably not to the same extent as primarily vascular insults. Blood–brain barrier dysfunction and decreased vascular integrity have been described in white matter disease (Sahlas et al., 2002). Microglial cell activation, related to the phagocytosis of myelin breakdown products, has also been described and is more active in periventricular white matter lesions, rather than deep white matter lesions (Simpson et al., 2007). Toxic effects on vascular permeability secondary to amyloid deposits and cerebral blood flow autoregulation have also been described in the pathogenesis of WMH (O’Sullivan et al., 2001).

Systemic vascular disorders and risk factors such as hypertension, diabetes and smoking are closely associated with WMHs and likely contribute to the above processes leading to their development. These risk factors and their biomechanical effects contribute to a multifactorial pathogenesis with visible lesions on imaging representing only the “tip of the iceberg.” This concept suggests that the hyperintensities on FLAIR sequences reflect just the most overt manifestation of underlying white matter damage, while a larger, diffuse burden of microstructural damage exists in the surrounding normal-appearing white matter, undectable by conventional imaging. Studies have shown that structural often extends beyond the visible WMH boundaries. For instance, O’Sullivan et al. (2002) identified reduced cerebral blood flow and structural damage in normal appearing white matter surrounding WMHs, suggesting hypoperfusion as an early feature in WMH development (O’Sullivan et al., 2002). Other studies have similarly reported microstructural changes radiating into peri-WMH tissue, indicating that white matter alterations are part of a diffuse process (Leritz et al., 2014; Maillard et al., 2014; Maniega et al., 2015).

Our most commonly observed WMHs were in the periventricular region with FA values most closely resembling normal appearing white matter, compared to lesions seen in the deep white matter. If a linear or progressive pattern of white matter disease is accepted, our finding supports the theory that periventricular white matter is more susceptible to microangiopathy. Our observation that deep white matter hyperintensities, both type A (low volume and high FA) and type C (high volume and low FA) were associated with poorer cognitive outcomes, supports the theory that deep white matter disease represents a more severe form of disease.

While our study sheds light on the complex relationship between WMH characteristics and cognitive outcomes, the vascular intricacies of the periventricular region warrant further investigation. Future studies exploring the hemodynamic aspects and perfusion dynamics specific to periventricular white matter are warranted and longitudinal studies with serial neuroimaging are needed to monitor the progression of WMHs and determine if lesions may regress with intervention.

## Conclusion

Our study provides insights into the complex relationship between WMH characteristics and cognitive decline, shedding light on potential novel phenotypes associated with differential cognitive outcomes, but the vascular intricacies of the periventricular region warrant further investigation. The identification of six distinct WMH phenotypes, coupled with a comprehensive consideration of demographic and health factors, improves understanding of the predictors of cognitive decline. Future longitudinal studies with serial neuroimaging are warranted to monitor the natural progression of WMHs and determine if interventions may lead to lesion regression with appropriate treatment and how this interacts with longitudinal cognitive outcomes.

While acknowledging inherent limitations, our findings contribute valuable insights into the multifaceted relationships between white matter integrity and cognitive outcomes, and paves the way for future efforts aimed at deciphering and mitigating the impact of WMHs on cognitive health in aging populations.

### Strengths and limitations

Our study, while offering valuable insights into the intricate relationship between white matter hyperintensity (WMH) phenotypes and cognitive decline, is characterised by both strengths and limitations that collectively contribute to the overall integrity of our findings.

### Strengths

Large, Representative Sample: The inclusion of a substantial MRI sample from a nationally representative population-based study enhances the external validity of our findings, allowing for broader generalisability to diverse groups of community-dwelling older adults.

Longitudinal Design: The longitudinal nature of our study spanning 6 years provides a temporal dimension to our analyses, enabling the exploration of cognitive decline over time and capturing changes in WMH phenotypes.

Advanced Lesion Phenotyping: Employing a data-driven approach for lesion phenotyping, considering both macrostructural and microstructural properties, adds depth to our characterisation of WMH subtypes. This nuanced approach enhances our understanding of the heterogeneity within WMHs.

### Limitations

**Cross-Sectional Nature of Lesion Phenotyping:** The identification of WMH phenotypes was based on cross-sectional analyses, limiting our ability to capture dynamic changes in lesion characteristics over time. Longitudinal studies with repeated imaging sessions could provide a more comprehensive understanding of the evolving nature of WMHs.

**Generalisability and Population Characteristics:** Our study focused on a specific cohort of community-dwelling older adults from a nationally representative population-based study. Generalisability to other demographic groups or clinical populations may be limited, and caution is advised when extrapolating findings to broader populations.

**Absence of a Control Group:** The absence of a control group composed of individuals without WMHs or with low lesion burden is a limitation of our study. Inclusion of a control group would have allowed for direct comparison between individuals with varied degrees of WMH burden and those with normal appearing white matter, lending strength to the subsequent findings and improving the validity of the study. In the absence of a control group it is difficult to isolate the effects of WMHs with regards to cognitive decline from other age related factors or comorbidities that could independently affect outcomes. In addition a control group would have provided a baseline trajectory of cognitive outcomes. Future research should incorporate an appropriately matched control group to enhance the outcomes and strengthen the causal relationship between WMH and cognitive decline.

**MR Acquisition Technique:** Our MR acquisition protocol utilises a slice thickness of 4 mm for both the FLAIR and diffusion sequences which represents a limitation of our study. A thickness of 4 mm reduces our spatial resolution and limits segmentation of smaller white matter lesions, and may contribute to partial volume effects (mixing of signals from adjacent tissue), compromising the ability to distinguish between smaller lesions. Thinner slices (2–3 mm ideally) would provide more accurate lesion segmentation. In addition the implementation of a multi-shell sequence would allow for more detailed characterisation of white matter microstructure through utilisation of multiple B value acquisitions. This would allow for diffusion kurtosis imaging and more complex analysis of white matter microstructure, enhancing the differentiation of our described white matter phenotypes. Furthermore, multi-shell imaging would mitigate the effect of partial volume errors. Adopting higher resolution images and a multi-shell technique would result in more accurate data.

Acknowledging both strengths and limitations, our study offers a nuanced exploration of the complex relationships between WMH characteristics and cognitive decline. The comprehensive approach, from advanced lesion phenotyping to consideration of diverse factors, contributes to a balanced interpretation of our findings. While our observations provide valuable insights, the identified limitations underscore the need for continued research refinement and external validation. Striking a balance between the strengths and limitations enhances the reliability and applicability of our study, paving the way for future investigations in this evolving field.

## Data Availability

The original contributions presented in the study are included in the article/supplementary material, further inquiries can be directed to the corresponding author.

## Footnotes

^1^ http://www.exploredti.com

^2^ https://www.mathworks.com/products/matlab.html

^3^ www.statistical-modelling.de/lst.html
